# Bacteriophage Receptor Binding Protein Based Assays for the Simultaneous Detection of *Campylobacter jejuni* and *Campylobacter coli*

**DOI:** 10.1371/journal.pone.0069770

**Published:** 2013-07-18

**Authors:** Muhammad A. Javed, Somayyeh Poshtiban, Denis Arutyunov, Stephane Evoy, Christine M. Szymanski

**Affiliations:** 1 Alberta Glycomics Centre and Department of Biological Sciences, University of Alberta, Edmonton, Alberta, Canada; 2 Department of Electrical and Computer Engineering, University of Alberta, Edmonton, Alberta, Canada; University of Illinois at Chicago College of Medicine, United States of America

## Abstract

*Campylobacter jejuni* and *Campylobacter coli* are the most common bacterial causes of foodborne gastroenteritis which is occasionally followed by a debilitating neuropathy known as Guillain-Barré syndrome. Rapid and specific detection of these pathogens is very important for effective control and quick treatment of infection. Most of the diagnostics available for these organisms are time consuming and require technical expertise with expensive instruments and reagents to perform. Bacteriophages bind to their host specifically through their receptor binding proteins (RBPs), which can be exploited for pathogen detection. We recently sequenced the genome of *C. jejuni* phage NCTC12673 and identified its putative host receptor binding protein, Gp047. In the current study, we localized the receptor binding domain to the C-terminal quarter of Gp047. CC-Gp047 could be produced recombinantly and was capable of agglutinating both *C. jejuni* and *C. coli* cells unlike the host range of the parent phage which is limited to a subset of *C. jejuni* isolates. The agglutination procedure could be performed within minutes on a glass slide at room temperature and was not hindered by the presence of buffers or nutrient media. This agglutination assay showed 100% specificity and the sensitivity was 95% for *C. jejuni* (n = 40) and 90% for *C. coli* (n = 19). CC-Gp047 was also expressed as a fusion with enhanced green fluorescent protein (EGFP). Chimeric EGFP_CC-Gp047 was able to specifically label *C. jejuni* and *C. coli* cells in mixed cultures allowing for the detection of these pathogens by fluorescent microscopy. This study describes a simple and rapid method for the detection of *C. jejuni* and *C. coli* using engineered phage RBPs and offers a promising new diagnostics platform for healthcare and surveillance laboratories.

## Introduction

*Campylobacter jejuni* and *Campylobacter coli* are the major foodborne pathogens that cause enteritis and diarrhea [1]. *Campylobacter* infection is generally self-limiting, however, it can be followed by post-infectious sequelae including the autoimmune neurological disorder, Guillain-Barré syndrome [2].

Poultry and swine are the natural hosts for *C. jejuni* and *C. coli* and are thus the main sources of campylobacteriosis through the ingestion of contaminated food [1]. Rapid detection of these pathogens is needed to avoid the spread through the food-chain and for quick treatment and effective control. Generally, campylobacters are isolated from any sample using filtration, culture enrichment and growth on selective media followed by identification using microscopy and biochemical assays. Though traditional culture based identification of bacterial isolates remains a gold standard, these conventional methods are laborious and time consuming. Over the last decade, many efforts have been made to develop tools for the rapid detection of *Campylobacter* species, especially *C. jejuni* and *C. coli*. After the publication of the first *C. jejuni* genome sequence in 2000 [3], most of the reported rapid detection methods are based on the polymerase chain reaction (PCR) including multiplex and real-time PCR [4], [5], [6] and require DNA isolation, removal of PCR inhibitors from the samples, expensive instruments and reagents, as well as significant expertise to run these tests. Serological typing of campylobacters is also a widely used technique however, cost of antibody production and their shorter shelf life are limitations for this method. Thus, there is a need for rapid, specific, cost-effective and simple methods for the simultaneous detection of the two major foodborne pathogens, *C. jejuni* and *C. coli.* Several new techniques have recently been described for the detection of these bacteria [4], [5], [6], [7], [8], [9], [10], [11], and any novel technique is expected to be superior in at least one major factor such as the specificity, rapidity, cost-effectiveness and/or ease of use.

Bacteriophages (or phages) have inherent host specificity and this specificity makes them excellent probes for identifying their target bacteria [12]. Phage typing schemes have been developed for many pathogens [13], [14], [15] including *Campylobacter* species [16], [17], [18]. Unfortunately, these schemes demand the laborious and time consuming preparation of bacterial cultures and may be limited by the problems associated with the production and storage of these phages. We have also shown that phages immobilized on solid surfaces can be utilized for the detection of bacterial pathogens without the necessity for traditional culturing [19], [20], [21]. However, like other detection methods, immobilized phage-based assays have their shortcomings. For example, desiccation of surface-immobilized phages severely impairs their binding efficiency, and over exposure of the bacterial cells to the phage particles results in the lysis and the eventual destruction of the captured bacteria being detected. These drawbacks of the immobilized phage based assays can be avoided by using phage receptor binding proteins (RBPs) instead of the whole phage particles [12], [22].

We recently sequenced the genome of the lytic *C. jejuni* phage NCTC12673 and identified a putative RBP, Gp047; previously annotated as Gp48 [23]. Gp047 does not share sequence homology with any characterized phage RBPs, but homologues are found in all sequenced campylobacter phages [24], [25], [26], [27], [28]. We have also shown that antibodies against Gp047 cross-react with native *Salmonella enterica* serovar Typhimurium phage P22 tail spike protein (TSP), but not denatured TSP, suggesting that these proteins have structural similarity [23]. In addition, Gp047 forms sodium dodecyl sulfate resistant oligomers [23], similar to the TSPs of *S*. Typhimurium phage P22 and *Shigella flexneri* phage SF6 [29], [30], and can specifically capture *C. jejuni* NCTC11168 cells when immobilized onto solid surfaces [11]. This ability of immobilized Gp047 to capture *C. jejuni* can also be exploited for the preconcentration of campylobacter cells before using more traditional methods for analyzing samples suspected to be contaminated with this organism.

Since Gp047 is an oligomer and is the largest protein encoded in the genome of NCTC12673 (152 kDa), its large size poses a challenge for expressing an active form of the protein in high yields. Here, we report the localization of the binding domain in Gp047 that allowed us to generate a shorter derivative that is one-quarter the size of the original protein, yet is capable of detecting both *C. jejuni* and *C. coli* and can be used in various diagnostic platforms. We developed a simple glass slide agglutination assay for pure cultures which enabled us to rapidly screen the host range of Gp047. We also report a fluorescent microscopy based method for the detection of *C. jejuni* and *C. coli* in mixed cultures. To our knowledge, this is the first report of a phage RBP-based assay for the simultaneous detection of these *Campylobacter* species.

## Results

### Localization of the Gp047 Binding Domain

**Figure 1:**
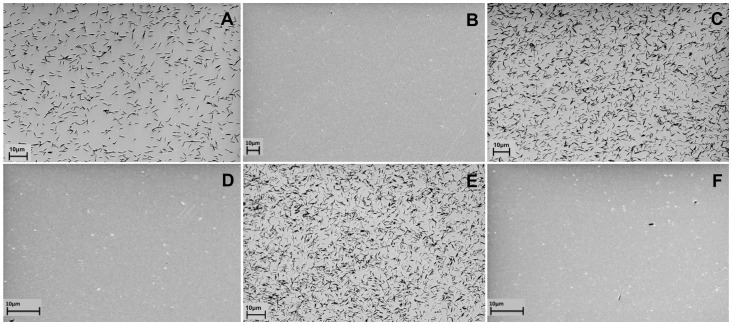
Scanning electron microscopy images of *Campylobacter jejuni* cells captured by immobilized Gp047 and its derivatives. GST-fused Gp047 (A), truncated N-Gp047 (B), C-Gp047 (C), NC-Gp047 (D), CC-Gp047 (E), and 4C-Gp047 (F) were immobilized onto gold-coated surfaces and incubated with *C. jejuni* NCTC11168 cells. Scale bar represents 10 µm.

The purified GST-Gp047 wildtype, N-Gp047 (residues 1–684), C-Gp047 (residues 681–1365), NC-Gp047 (residues 681–1041), CC-Gp047 (residues 1040–1365), 3C-Gp047 (residues 1040–1204) and 4C-Gp047 (residues 1204–1365) derivatives were immobilized onto solid surfaces and were tested for their ability to capture *C. jejuni* NCTC11168 cells. The NCTC11168 cells were captured by Gp047 wildtype, C-Gp047and CC-Gp047, but not by N-Gp047 or NC-Gp047 indicating that the host binding domains were localized in the C-terminal quarter of Gp047 **(** **Fig. 1** **)**. Further truncation of CC-Gp047 into 3C-Gp047 and 4C-Gp047 resulted in the complete loss of binding **(** **Fig. 1**, 3C-Gp047 not shown**)**.

**Figure 3:**
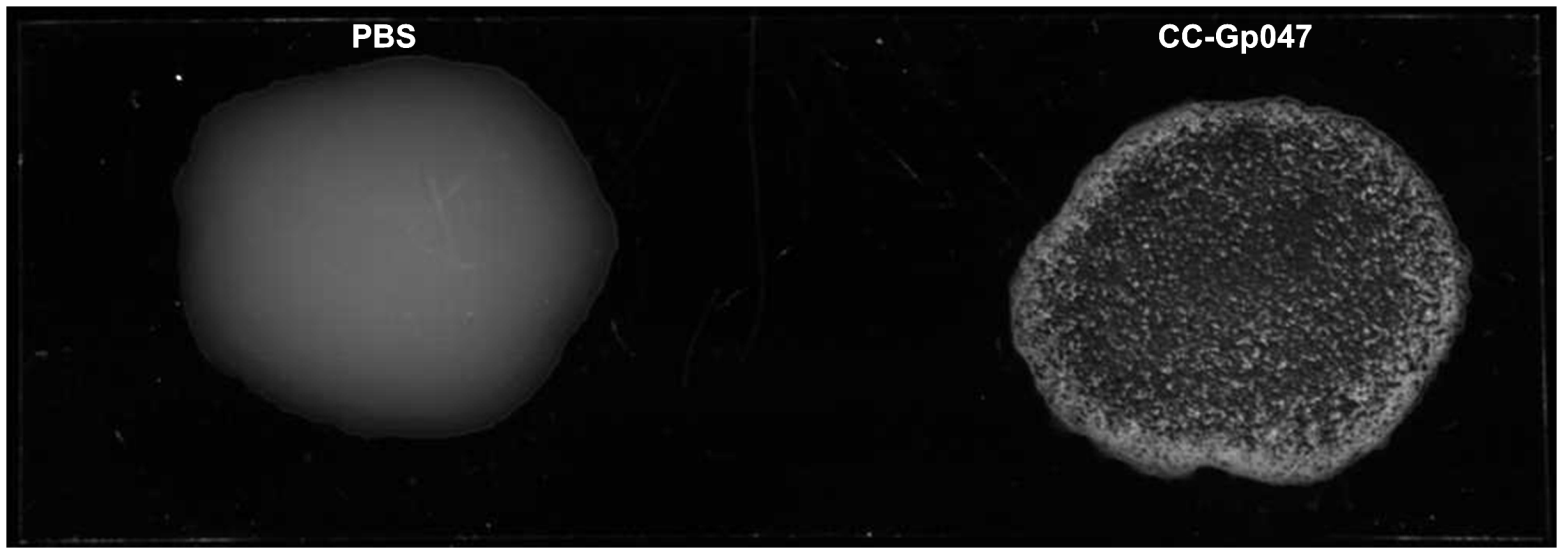
Agglutination of *Campylobacter jejuni* NCTC11168 cells with CC-Gp047. *C. jejuni* NCTC11168 cells were agglutinated with CC-Gp047 but not with PBS alone.

### Confirmation of Host Binding Specificity

**Figure 2:**
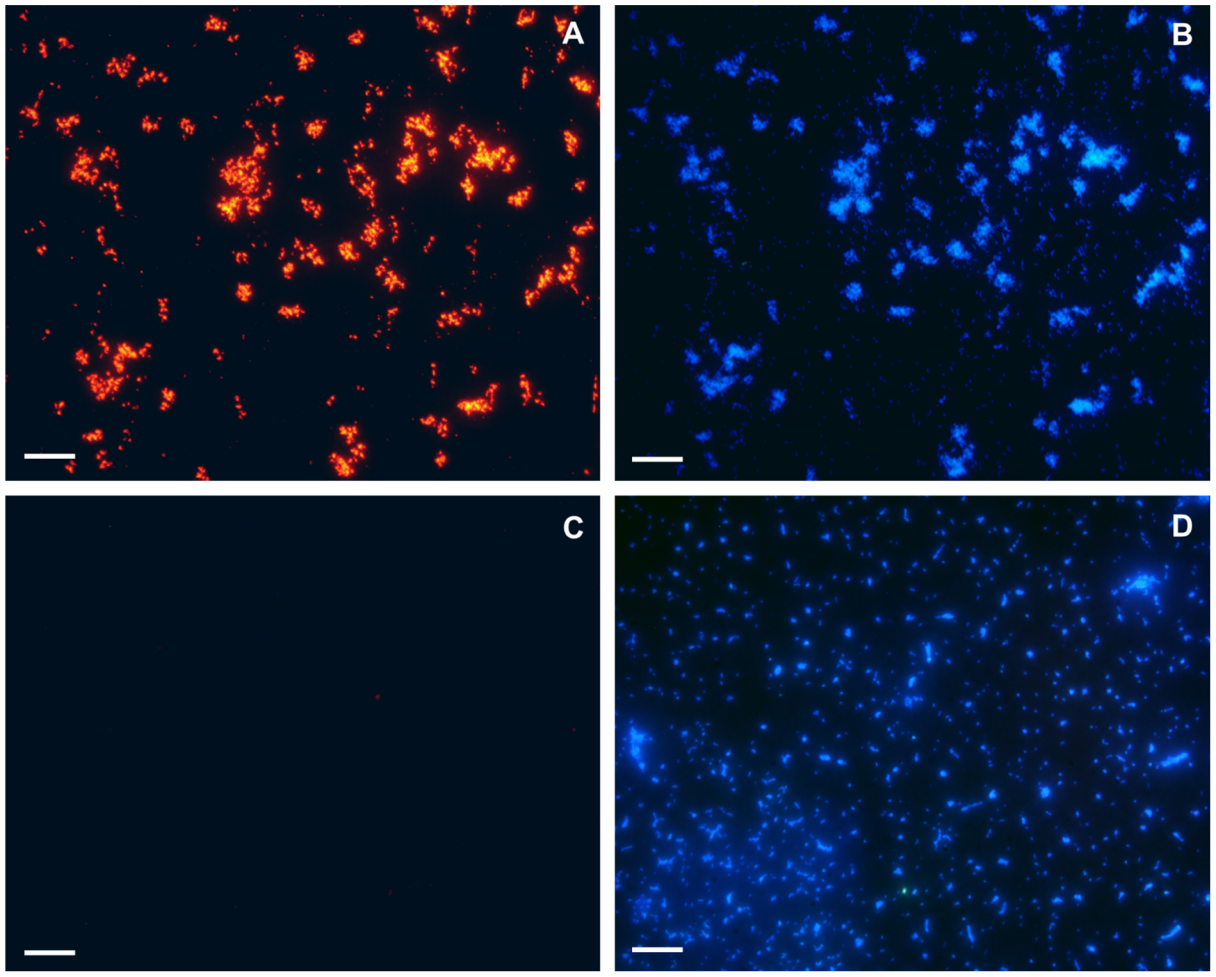
Immunofluorescence microscopy images of *Campylobacter jejuni* cells after incubation with CC-Gp047. CC-Gp047 showed binding to *C. jejuni* NCTC11168 (A) but not to *S. enterica* serovar Typhimurium (C). DAPI stained *C. jejuni* NCTC11168 (B) and *S.* Typhimurium (D). Scale bar represents 10 µm.

Host binding specificity was also tested through immunolabeling of the bacterial cells with CC-Gp047 followed by detection with anti-Gp047 antibodies and Alexa Fluor®546 conjugated secondary antibodies. Fluorescent microscopy of the samples showed that CC-Gp047 binds specifically to *C. jejuni* NCTC11168, but not to *S.* Typhimurium which was used as a negative control **(** **Fig. 2** **)**. The DNA intercalating dye, DAPI, stained both *C. jejuni* as well as *S.* Typhimurium cells.

### Agglutination Assay

An agglutination assay combining *C. jejuni* NCTC11168 cells with CC-Gp047 formed aggregates within one minute **(** **Fig. 3** **)**. It has previously been demonstrated that *C. jejuni* is also capable of autoagglutination [31]. To rule out the possibility that bacteria were autoagglutinating, we always included a control suspension of the respective bacterial strain in PBS without CC-Gp047. The results showed that agglutination was caused by CC-Gp047 and was not due to autoagglutination since control suspensions lacking CC-Gp047 did not show agglutination. Other derivatives of Gp047 including N-Gp047, NC-Gp047, 3C-Gp047 and 4C-Gp047 did not cause agglutination of *C. jejuni* NCTC11168.

Next, the bacterial cells were suspended in different growth media (MH, BHI, LB, and NCZYM) as well as in different buffers (phosphate buffer pH 7.4, HEPES buffer pH 7.4, normal saline, Tris-HCl buffer pH 7.5 and 5% BSA in PBS) to determine if the agglutination assay can be performed in different salts and complex media. The assay showed the same results in all tests.

To test if bacterial cell growth phase or viability has an effect on the agglutination assay, we tested *C. jejuni* NCTC11168 cells collected after 18 and 62 hours growth containing cells in different growth states with the majority in the viable but non-culturable state in the 62 hour culture. Both preparations showed agglutination with CC-Gp047. In addition, formalin killed *C. jejuni* NCTC 11168 cells showed agglutination with CC-Gp047. These results suggest that the growth state has no influence on the recognition of the bacterial cells by this protein and the agglutination assay does not differentiate between live or dead cells.

We also tested the stability and shelf-life of the protein by testing its ability to agglutinate *C. jejuni* NCTC11168 at different time points after purification. CC-Gp047 was able to consistently agglutinate the bacterial cells up to the latest tested time point of 18 months after being stored at 4°C (data not shown).

### Specificity and Sensitivity of the Assay

To check the host range of CC-Gp047, we tested several campylobacter strains isolated from a variety of sources including symptomatic and asymptomatic human patients, animal and animal products, environment and laboratory strains. In total, 40 strains of *C. jejuni*, 19 strains of *C. coli*, and representative strains of other *Campylobacter* species including *C. fetus* subsp. *fetus*, *C. fetus* subsp. *venerealis*, *C. lari*, *C. upsaliensis* and *C. concisus* were tested. In addition, we tested two strains of the related human pathogen *Helicobacter pylori,* two laboratory strains of *E. coli,* and *S.* Typhimurium. In total 38 out of 40 (95%) isolates of *C. jejuni* showed agglutination when mixed with CC-Gp047 and unexpectedly 17 out of 19 (90%) isolates of *C. coli* were also agglutinated in the presence of CC-Gp047 **(** **Table 1** **)**. The assay showed 100% specificity for *C. jejuni* and *C. coli* as none of the other *Campylobacter* species, *H. pylori, E. coli* or *S.* Typhimurium showed agglutination with CC-Gp047 **(** **Table 1** **).** This confirmed that the assay can be used for the specific and simultaneous detection of *C. jejuni* and *C. coli*. Interestingly, CC-Gp047 showed a much broader host range in the agglutination assays compared to the lytic host range of phage NCTC 12673 from where Gp047 was derived from.

**Table 1:** List of bacterial strains used and results of agglutination assay.

Organism	Strains	Sources/disease	Agglutination
*C. jejuni*	Cj60^a^, 81–176* b, NCTC12517^a^, HS:3^i^, **NCTC11168** c, NCTC11168H^c^, RM1244^d^	Human/Enteritis	Positive
	Cj31467, Cj31481, Cj31485, Cj32787, Cj33084, Cj33106^e^	Human/Asymptomatic	Positive
	RM1221* g, RM1184, RM1216, RM1285, RM1850, RM3668, RM4162, Cj120, Cj150, Cj160, Cj178, Cj198, Cj206, Cj207, Cj220, Cj230, Cj11919, Cj11974, **Cj12567** a **, NCTC12658,** NCTC12659, NCTC12660*, NCTC12661, NCTC12663*, NCTC12664, NCTC12665 a	Animal/Animal products	Positive
	Cj212, Cj11848	Animal/Animal products	Negative
*C. coli*	ROH1, ROH2, Cc167, Cc345, Cc423, Cc456^a^	Frozen poultry	Positive
	Cc11, Cc145, Cc253, Cc255, Cc525, Cc700, NCTC12532*, RM1515^a^, RM2228* f	Animal/animal products	Positive
	RM1891^g^	Chicken farm	Positive
	RM1900^g^	Swine farm	Positive
	VC167, Cc463^a^	Human, Animal	Negative
*C. lari*	RM2100^f^	Human	Negative
*C. fetus fetus*	NCTC10482*	Sheep/Abortion	Negative
*C. fetus venerealis*	NCTC10354*	Cow/Venereal disease	Negative
*C. concisus*	NCTC11485	Human/Periodontitis	Negative
*C. upsaliensis*	RM3195^f^	Human/Guillain-Barré syndrome	Negative
*H. pylori*	J99^h^, G27^i^	Human	Negative
*E. coli*	DH5α, BL21	Invitrogen®	Negative
*S. enterica*	serovar Typhimurium	ATCC 19585	Negative

a McNally, D. J.*et al*. 2007 [41],

b Black, R. E.*et al*. 1988 [42],

c Parkhill, J.*et al*. 2000 [3],

d Miller, W. G.*et al*. 2005 [43],

e Champion, O. L.*et al*. 2005 [44],

f Fouts, D. E.*et al.* 2005 [45],

g Mandrell, R.E. *et al.* 2005 [46],

h Alm, R. A.*et al*. 1999 [47],

i Covacci, A.*et al*. 1993 [48].

Strains highlighted in bold were also tested for lysis by *Campylobacter* phage NCTC12673. The asterisks indicate strains unable to be lysed by the phage.

### Direct Detection of *C. jejuni* and *C. coli* using EGFP-fused CC-Gp047

**Figure 4:**
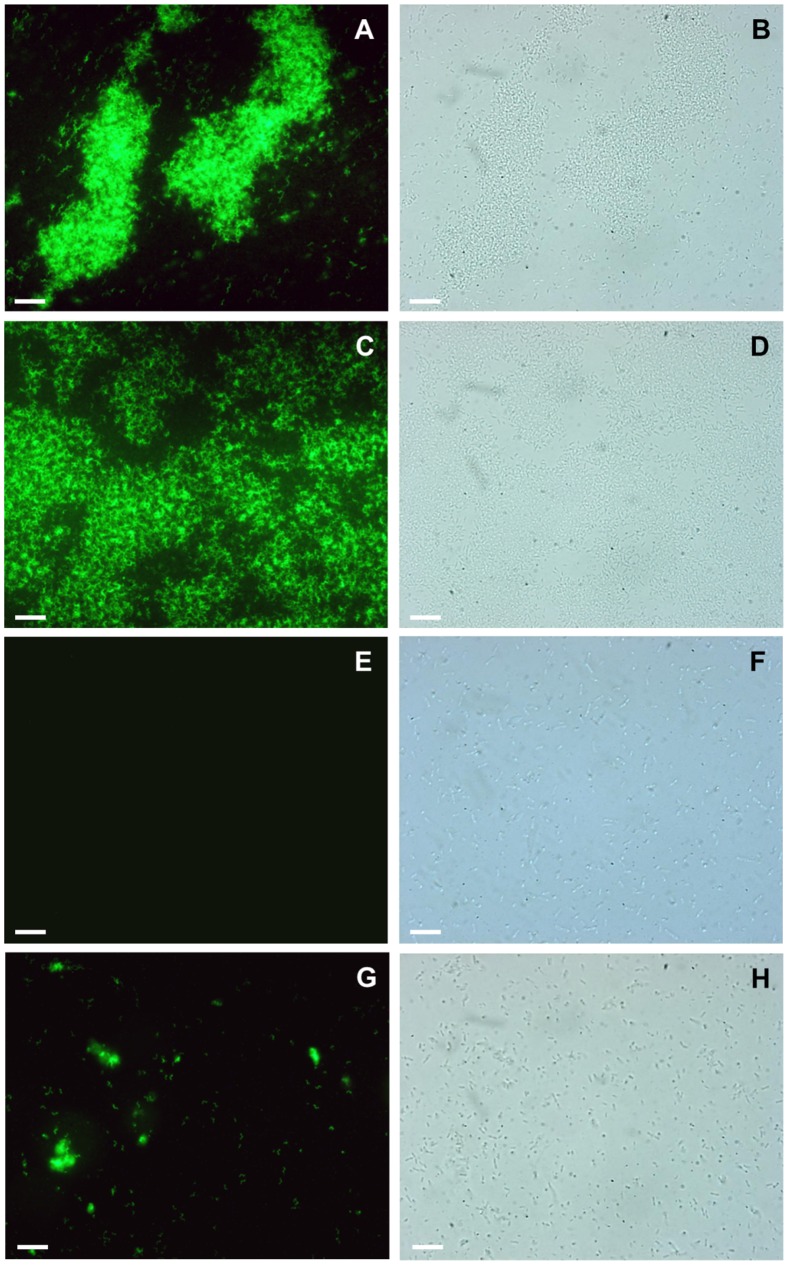
Fluorescent microscopy images of mixtures of *Escherichia coli*, *Campylobacter jejuni* and/or *Campylobacter coli* cells after incubation with EGFP_CC-Gp047. Bacterial cell suspensions were incubated with EGFP_CC-Gp047 and imaged by both fluorescence microscopy (A, C, E, G) and bright field microscopy (B, D, F, H). EGFP_CC-Gp047 bound specifically to *C. jejuni* NCTC11168 (A, B), *C. coli* RM2228 (C, D) but not to *E. coli* DH5α (E, F). EGFP_CC-Gp047 also showed specific binding to *C. jejuni* NCTC11168 in a mixed suspension of *C. jejuni* NCTC11168 and *E. coli* DH5α at a ratio of 1∶25 (G, H). Scale bar represents 10 µm.

For the detection of *C. jejuni* and *C. coli* cells in mixed cultures, we generated CC-Gp047 fused with the fluorescent reporter protein, EGFP. Then *C. jejuni* NCTC11168, *C. coli* RM2228 and *E. coli* DH5α cell suspensions were individually incubated with chimeric EGFP_CC-Gp047, washed and observed under a fluorescent microscope. The *C. jejuni* and *C. coli* cells were detected as green fluorescing cells **(** **Fig. 4:** **A, C)** indicating the binding of EGFP_CC-Gp047 while the *E. coli* DH5α cells **(** **Fig. 4:** **E)** did not show fluorescence, as expected. This confirmed that CC-Gp047 maintains its binding activity and specificity when expressed as a chimeric EGFP_CC-Gp047 protein. We then prepared mixed cultures of *C. jejuni* NCTC11168 and *E. coli* DH5α by mixing the respective cell suspensions at a ratio of 1∶25 (cell numbers) and tested if EGFP_CC-Gp047 can be used to specifically detect *C. jejuni* from a mixed culture. Only *C. jejuni* cells were specifically labelled **(** **Fig. 4:** **G)** in the mixed culture. We also repeated the detection assay using whole cell lysates of IPTG induced *E. coli* C43 cells expressing EGFP_CC-Gp047. Fluorescent microscopy of the bacterial cells exposed to the EGFP_CC-Gp047 containing lysate still showed the effective labelling of *C. jejuni* NCTC11168 and *C. coli* RM2228, but not *E. coli* DH5α cells (not shown) suggesting that purification of the EGFP_CC-Gp047 is not necessary for the detection assay. This further increases the ease of EGFP_CC-Gp047 use for the detection of *C. jejuni* and *C. coli*.

## Discussion

*C. jejuni* and *C. coli* are the major causative agents of campylobacteriosis and are responsible for more than 95% of human cases. We exploited the specificity of Gp047, the putative RBP of *C. jejuni* phage NCTC12673, to develop assays for the rapid detection of *C. jejuni* and *C. coli.* Gp047 does not exhibit any homology with other characterized phage RBPs using the basic alignment search tool (blast.ncbi.nlm.nih.gov/) or Pfam searches (http://pfam.sanger.ac.uk/). Thus, it was difficult to predict the location of the binding domains in this protein. Interestingly, the C-terminal part of the Gp047 is conserved in all the campylobacter phages where genome sequence data is available [28] and this is consistent with the results from this study which revealed that the host binding domain was also localized in the C-terminal part of this protein. The host binding domains of other phage RBPs have also been reported to be localized in the C-terminal [32], [33], [34] or the central portion of the protein [35] while the N-terminus is generally involved in the attachment of the tail receptor binding elements (either long fibers or short tail spikes) to the phage particle body [32], [33], [34]. The role of the N-terminus of Gp047 is yet not known, however, like most other tailed-phage RBPs, the C-terminus of this protein was found to be responsible for bacterial host recognition.

Receptor binding proteins of many phages including the TSPs of the well-characterized *S.* Typhimurium phage P22 and *S. flexneri* phage SF6 form trimers [29], [30]. It was shown that Gp047 also forms oligomers [23]. Similar to the full-length Gp047, CC-Gp047 also formed trimers and higher order SDS-resistant oligomers (data not shown). This observation explains the ability of CC-Gp047 to agglutinate *C. jejuni* cells.

Interestingly, Gp047 was found to agglutinate a broader spectrum of bacterial strains compared to the lytic range of phage NCTC12673. A broader host spectrum was also observed for other phage-derived proteins compared to their host phage. For example, the recombinant cell binding domain (CBDP40) derived from listeria phage P40 showed broader binding activity than phage P40 [36]. Similarly, the recombinant phage lysin (LysK) derived from staphylococcus phage K had broader lytic activity compared to whole phage K [37].

Our RBP-based agglutination assay is very rapid and requires only a glass slide so the method can be used to complement any of the traditional methods. Indeed, this agglutination assay showed 100% specificity for *C. jejuni* and *C. coli* and 95% and 90% sensitivity for *C. jejuni* and *C. coli*, respectively which is better or comparable to other *Campylobacter* species detection methods. Besséde *et al*. compared conventional campylobacter culture methods to two molecular biology methods: an in-house real-time PCR and a multiplex PCR named Seeplex Diarrhea ACE Detection, and three immunoenzymatic methods: Premier Campy, RidaScreen Campylobacter, and ImmunoCard Stat!Campy. The authors found that the specificity of all these methods was in the range of 95 to 100% while sensitivity was 80% for the in-house PCR, 88% for the multiplex PCR, 95% for the Premier Campy and 91% for the immunoCard method [38]. Poppert *et al*. used a fluorescent *in situ* hybridization technique to detect *Campylobacter* species and observed a sensitivity of 90% and 97% for *C. jejuni* and *C. coli*, respectively [39]. One can conclude that the Gp047 RBP-based detection method offers comparable levels of sensitivity and specificity while lacking the drawbacks associated with the aforementioned diagnostic procedures (e.g. excessive labor and/or time consumption as well as the need for expensive equipment and/or reagents).

CC-Gp047 also retains its activity after storage at 4°C indicating that the protein remains stable during prolonged storage. And, bacteria can be resuspended in any of the most commonly used growth media and buffers for the agglutination assays providing greater flexibility for the users. The results demonstrated that different salts, salt concentrations as well as complex growth media do not influence the binding of Gp047 to bacterial cells. We have also developed an assay to separate and enrich *C. jejuni* cells from complex food matrices such as milk and chicken broth spiked with the bacteria using CC-Gp047 immobilized onto magnetic beads (S. Poshtiban *et al*., submitted). Furthermore, CC-Gp047 can be used in any platforms currently using antibodies and thus can potentially be used for bacterial detection from single isolated colonies in microbiological laboratories. For the direct detection of *C. jejuni* and *C. coli* from a sample containing a mixture of bacterial species, CC-Gp047 was expressed as a chimeric protein fused with a reporter fluorescent protein. The EGFP_CC-Gp047 chimera recognized both *C. jejuni* and *C. coli* cells and was able to specifically detect these cells even when they were in low numbers or in a mixed culture. Like other fluorescence based assays, the presence of fluorescence quenchers or autofluorophores in the sample may affect the efficiency of this assay. If required, these potential inhibitors can be removed by centrifugation or magnetic bead enrichment techniques.

Therefore we describe two rapid, simple and specific assays that provide proof-of-principle that a short derivative of the campylobacter phage receptor protein, CC-Gp047, can be used for the simultaneous detection of the major pathogenic *Campylobacter* species (*C. jejuni* and *C. coli*). These assays are not technically demanding and do not need special instrumentation, with the exception of a fluorescent microscope for the EGFP assays. Furthermore, since bacteriophage abundance is estimated at 10^31^ particles worldwide, they represent a limitless resource for the development of novel diagnostic platforms against any bacterial host.

## Materials and Methods

### Bacterial Strains and Growth Conditions

C. *jejuni* and *C. coli* strains were routinely grown on Mueller Hinton (MH) agar (Difco) at 37°C under microaerobic conditions (85% N_2_, 10% CO_2_, and 5% O2). Other *Campylobacter* species and *H. pylori* strains were grown on MH agar supplemented with horse blood (5%, v/v). S. *enterica* serovar Typhimurium was grown on MH agar under aerobic conditions. *Escherichia coli* strains were grown on Luria Bertani (LB) agar supplemented with ampicillin, kanamycin or chloramphenicol at a final concentration of 100, 50 or 20 µg/ml, respectively, where needed. The list and sources of bacterial strains used in this study are given in **Table 1**.

### Localization of Host Binding Domains in Gp047

To localize the host binding domains, truncated versions of Gp047 were expressed as GST-fusions using the pGEX 6P-2 vector (GE Healthcare) and *E. coli* BL21 cells (Invitrogen) unless otherwise mentioned. The lists of plasmids and primers used in this study are given in **Table 2** and **Table 3**, respectively. Initially *gp047* DNA fragments encoding for the N- (residues 1–684) and C-terminal (residues 681–1365) parts of Gp047 were amplified from pGEX_*gp047* [23] using primer pairs CS710/CS711 and CS712/CS713, respectively. Each amplicon was digested with *Eco*RI and *Xho*I and ligated with the *Eco*RI/*Xho*I double digested pGEX 6P-2 vector. The resulting vectors pGEX_*ntgp047* and pGEX_*ctgp047* were transformed into *E. coli* DH5α and then BL21; colonies were selected on LB agar supplemented with ampicillin. The in-frame cloning of the DNA fragments was confirmed by restriction analysis and nucleotide sequencing performed at the Molecular Biology Service Unit, Biological Sciences Department, University of Alberta.

**Table 2:** List of plasmids used in this study.

Plasmid	Description	Source or reference
pGEX 6P-2	Glutathione-S-transferase (GST) fused protein expression vector, ampicillin resistance marker, *tac* promoter	GE Healthcare
pGEX_*gp047*	*gp047* in frame cloned into multiple cloning site of pGEX 6P-2 for expression of GST-fused Gp047	[23]
pGEX_*ntgp047*	Expression construct of GST-fused N-Gp047 in pGEX 6P-2	This study
pGEX_*ctgp047*	Expression construct of GST-fused C-Gp047 in pGEX 6P-2	This study
pGEX_*ncgp047*	Expression construct of GST-fused NC-Gp047 in pGEX 6P-2	This study
pGEX_*ccgp047*	Expression construct of GST-fused CC-Gp047 in pGEX 6P-2	This study
pGEX_3cgp047	Expression construct of GST-fused 3C-Gp047 in pGEX 6P-2	This study
pGEX_*4cgp047*	Expression construct of GST-fused 4C-Gp047 in pGEX 6P-2	This study
pET28_*ccgp047*	Expression construct of 6xHis -fused CC-Gp047 in pET28a	This study
pET28_*egfp-ccgp047*	Expression construct of 6xHis-fused CC-Gp047 in pET28a	This study
pET28a	Protein expression vector, 6xHis tag, kanamycin resistance marker, T7 promoter	Invitrogen

**Table 3:** List of primers used in this study.

Primer	Nucleotide sequence (5′ –3′)
**CS710**	GCCCCTGGGATCCCCAGGAATTCC
**CS711**	* AAGGCTCGAGTTATAGAACTGCAATAACTTTAGTTCCACTAG
**CS712**	* AAGGAATTCCCGCAGTTCTAACTTCTGATAACAGTAAAACAAATG
**CS713**	CAGATCGTCAGTCAGTCACGATGC
**CS716**	* AAGGAATTCCCTCCAAACAAGTTGATAATATATCTTTTCA
**CS717**	* AAGGCTCGAGGGAGCTATTTATATCAACTTGGTAATAAGG
**CS725**	* AAGGCTCGAGTCCTATTTTAACACCGCTCATACA
**CS726**	* AAGGAATTCCCGGATTTGATAACGGATATGCTTCT
**CS1005**	* ATATTAGCCATATGGTGAGCAAGGGCGAGGAG
**CS1006**	* ATATGGATCCCTTGTACAGCTCGTCCATGCC

* Additional nucleotides and *Bam*HI (ggatcc), *EcoRI* (gaattc), *NdeI* (catatg), or *XhoI* (ctcgag) restriction site at 5′ end of the primers are underlined, where applicable.

The C-terminal half of Gp047 was further truncated into NC-Gp047 (residues 681–1041) and CC-Gp047 (residues 1040–1365); their encoding DNA fragments were amplified using primer pairs CS710/CS717 and CS716/CS713, respectively. The amplicons encoding for NC-Gp047 and CC-Gp047 were double digested with *Eco*RI and *Xho*I and cloned into pGEX 6P-2 as described above. The resulting vectors pGEX_*ncgp047* and pGEX_*ccgp047* were confirmed by restriction analysis and nucleotide sequencing.

Further truncations of CC-Gp047 were generated creating 3C-Gp047 (residues 1040–1204) and 4C-Gp047 (residues 1204–1365); DNA fragments encoding for 3C-Gp047 and 4C-Gp047 were amplified from pGEX_*ccgp047* using primer pairs CS710/CS725 and CS726/CS713, respectively. PCR products were digested with *Eco*RI and *Xho*I and were cloned into pGEX 6P-2 as described above. The resulting vectors pGEX_*3cgp047* and pGEX_*4cgp047* were analysed by restriction analysis and nucleotide sequencing.

### Construction of EGFP-fused CC-Gp047

The EGFP-fused CC-Gp047 was expressed in the pET28a (Invitrogen) vector in which EGFP was fused at the N-terminal end of CC-Gp047 and has a His-tag at the N-terminal end. The *ccgp047* was cut from pGEX_*ccgp047* using *Bam*HI and *Xho*I and cloned into pET28a pre-digested with *Bam*HI and *Xho*I; the resulting vector pET28_*ccgp047* was transformed into *E. coli* DH5α and then into *E. coli* C43 (derivative of *E. coli* BL21 [DE3]) cells. The *egfp* was amplified from pBAD/HisB_*egfp* (a gift from Robert E. Campbell, Chemistry Department, University of Alberta) with primers CS1005 and CS1006. The PCR products were digested with *Nde*I and *Bam*HI and cloned into *Nde*I and *Bam*HI digested pET28_*ccgp047.* The resulting plasmid pET28_*egfp*-*ccgp047* was transformed into *E. coli* C43 cells and colonies were selected on LB agar supplemented with kanamycin. The in-frame cloning was confirmed by nucleotide sequencing of the constructs.

### Protein Expression and Purification

The full GST-Gp047 protein and GST-fused Gp047 derivatives were expressed in *E. coli* BL21 cells and the proteins were purified as described previously [23].

The EGFP-fused CC-Gp047 was expressed in *E. coli* C43 cells. The bacterial cells harbouring pET28_*ccgp047* or pET28_*egfp-ccgp047* were grown at 30°C in 2xYE broth while shaking at 200 rpm. When the culture reached an OD_600_ of 0.3, the cells were induced with 0.5 mM isopropyl β-D-thiogalactopyranoside (IPTG) and shaken for 6 hours. The culture was then stored overnight at 4°C to allow complete maturation of the EGFP chromophore. The cells were harvested by centrifugation at 10,000×g for 15 minutes, resuspended in buffer A (20 mM sodium phosphate, 500 mM NaCl, 30 mM imidazole, pH 7.4) supplemented with the protease inhibitor cocktail (Roche) and were lysed by using a cell disrupter (Constant Systems, UK). The lysate was centrifuged at 17400×g for 30 minutes followed by filtration through 0.2 µm syringe filters (Millipore). The protein was purified by immobilized metal ion affinity chromatography using a nickel-nitrilotriacetic acid (Ni-NTA) agarose (Qiagen) column according to the manufacturer’s protocol. The captured protein was eluted from the Ni-NTA column using buffer B (20 mM sodium phosphate, 500 mM NaCl, 500 mM imidazole, pH 7.4) and dialyzed against 4 L of phosphate buffered saline (pH 7.5), with two buffer changes. The protein concentration was determined by measuring sample absorbance at 280 and 260 nm. The protein purity was determined by 12% sodium dodecyl sulfate-polyacrylamide gel electrophoresis followed by Coomassie blue staining.

### Bacterial Capture by Immobilized GST-Gp047 and Derivatives

The ability of Gp047 and its derivatives to capture *C. jejuni* was assayed as described previously [11]. Briefly, the gold substrates were washed sequentially in acetone, isopropyl alcohol, ethanol, and water for 5 min each to clean the surface prior to functionalization. The clean gold chips were incubated in a 2 mg/ml solution of glutathione in PBS for 1 h on an orbital shaker at 1000 rpm. The formation of a gluthathione self-assembled monolayer (GSH-SAM) ensures oriented attachment of proteins, resulting in a 3-fold improvement in bacteria capture density [11]. GST-Gp047 or its derivatives were immobilized onto the surfaces by incubating the GSH-SAM gold chips with a 5 µg/ml solution of GST-Gp047 in PBS for 1 h at room temperature, followed by similar incubation with a 1 mg/ml solution of bovine serum albumin to block the free substrate surface. The gold substrates were exposed to *C. jejuni* NCTC11168 at 10^9^ CFU/ml in PBS for 30 min at room temperature. The substrates were washed, fixed, dried, and imaged using a scanning electron microscope (LEO 1430). The bacterial binding density was estimated from the images by using the cell counter plugin of ImageJ software (National Institutes of Health).

### Immunostaining of CC-Gp047 Bound to Bacterial Cells

Bacterial cells that were grown overnight were resuspended in 50 mM Tris-HCl buffer (pH 7.5) and added to a clean glass slide. After air drying, the bacteria were fixed with 5% glutaraldehyde for 5 minutes and then blocked with blocking buffer (5% BSA in 50 mM Tris-HCl buffer, pH 7.5). The slide was put into purified GST-fused CC-Gp047 (10 µg/ml final concentration) solution prepared in blocking buffer and incubated for one hour followed by washing 3x with Tris-HCl buffer. The slide was then put into rabbit anti-Gp047 antibody (1∶1000 dilution) solution [23] and incubated for one hour and then washed 3x with Tris-HCl buffer. The slide was then exposed to 4′,6-diamidino-2-phenylindole (DAPI) 1∶1000 diluted from a 2 mg/ml stock solution and Alexa Fluor® 546 conjugated goat anti-rabbit IgG antibodies (Invitrogen) 1∶500 diluted in blocking buffer for one hour, followed by washing 3x with Tris-HCl buffer and air drying with minimal exposure to light. The slide was then observed under a Leica DMRXA fluorescent microscope.

### Agglutination Assay

Bacterial overnight growth was harvested from agar plates and resuspended in PBS to an OD_600_ ∼1.5. A 50 µl aliquot of the cell suspension was mixed with 1 µl of CC-Gp047 (0.4 mg/ml) on a glass slide and swirled. Agglutination began to appear in the first minute, but the slide was swirled for up to 3 minutes in negative tests. The agglutination assay was also done using other derivatives of Gp047 including NC-Gp047, 3C-Gp047 and 4C-Gp047. Specificity or the true negative rate that measures the proportion of negatives which are correctly identified, and sensitivity or the true positive rate that measures the proportion of actual positive samples which are correctly identified were calculated using the formula, specificity = d/(b+d)×100, and sensitivity = a/(a+c)×100 where a: true positive; b: false positive; c: false negative and d: true negative.

The agglutination assay was performed with cells suspended in different growth media including MH, brain heart infusion, LB, and NZCYM broth as well as in phosphate buffer pH 7.4, HEPES buffer pH 7.4, saline (0.9% w/v sodium chloride in distilled water), Tris-HCl buffer pH 7.5, and 5% BSA in PBS.

The effect of bacterial cell viability on the agglutination assay was tested by using formalin killed cells. *C. jejuni* NCTC 11168 cells were suspended in PBS containing 2.5% formalin for 15 minutes, then cells were washed with PBS and finally resuspended in PBS.

### Phage Lytic Assay

The phage lytic assay was performed as previously described [40] with minor modifications. Briefly, overnight bacterial growth was resuspended in brain heart infusion broth supplemented with 1 mM CaCl_2_ and 10 mM MgSO_4_ and incubated for 4 hours. Then the OD_600_ of the culture was adjusted to 0.2 and 50 µl of the suspension was mixed with 5 ml NZCYM (0.6% w/v) overlay agar at 45°C. The mixture was then poured onto NZCYM agar plates. After solidification, the plates were spotted with 10 µl of phage suspensions. The spots were allowed to dry at room temperature before incubation for 24 h at 37°C under microaerobic conditions.

### Detection of Bacteria using Chimeric EGFP_CC-Gp047

Bacterial growth was harvested from agar plates and the OD_600_ was adjusted to 0.3 in 50 mM Tris-HCl buffer (pH 7.5); 5 µl purified EGFP-Gp047 (1.0 mg/ml) was mixed with 200 µl of the cell suspension and incubated for 20 minutes at room temperature on a shaker. The cells were washed three times with 50 mM Tris-HCl buffer by centrifugation at 5000×g using a bench-top centrifuge for 2 minutes to remove unbound protein and finally resuspended in 100 µl buffer. The cells were then observed under a Leica DMRXA fluorescent microscope.

In addition to purified EGFP_CC-Gp047, the bacterial cells were also stained using whole cell lysates of IPTG induced *E. coli* C43 cells expressing EGFP_CC-Gp047. For the preparation of the cell lysate, *E. coli* C43 cells with pET28_*egfp*-*ccgp047* were grown in 2xYT broth and were induced by IPTG as described above. The induced cells were resuspended in 50 mM Tris-HCl buffer containing a protease inhibitor cocktail (Roche) and were sonicated. After centrifugation, the lysate was filter sterilized through a 0.2 µm syringe-driven filter and stored at 4°C overnight to allow complete maturation of the EGFP chromophore. Then 7 µl of the lysate (protein concentration 1.6 mg/ml) was mixed with 200 µl bacterial cell suspension and incubated for 20 minutes at room temperature on a shaker, washed 3X and then resuspended in 100 µl Tris-HCl buffer.
